# Livestock First Reached Southern Africa in Two Separate Events

**DOI:** 10.1371/journal.pone.0134215

**Published:** 2015-08-21

**Authors:** Karim Sadr

**Affiliations:** School of Geography, Archaeology and Environmental Studies, University of the Witwatersrand, Johannesburg, South Africa; Universidade do Algarve, PORTUGAL

## Abstract

After several decades of research on the subject, we now know when the first livestock reached southern Africa but the question of how they got there remains a contentious topic. Debate centres on whether they were brought with a large migration of Khoe-speakers who originated from East Africa; or whether the livestock were traded down-the-line among hunter-gatherer communities; or indeed whether there was a long history of diverse small scale population movements in this part of the world, one or more of which ‘infiltrated’ livestock into southern Africa. A new analysis of the distribution of stone toolkits from a sizeable sample of sub-equatorial African Later Stone Age sites, coupled with existing knowledge of the distribution of the earliest livestock remains and ceramics vessels, has allowed us to isolate two separate infiltration events that brought the first livestock into southern Africa just over 2000 years ago; one infiltration was along the Atlantic seaboard and another entered the middle reaches of the Limpopo River Basin. These findings agree well with the latest results of genetic research which together indicate that multiple, small-scale infiltrations probably were responsible for bringing the first livestock into southern Africa.

## Introduction

The first European explorers and settlers on the west and south coasts of southern Africa obtained sheep and cattle from people who spoke a Khoe language, and for long we assumed that the Khoe-speakers had originally migrated into southern Africa *en masse* bringing the first livestock with them[1–3]. With direct radiocarbon dating of ancient livestock bones from several archaeological sites (Table 1), we are now certain that the earliest sheep and cattle appeared in southern Africa around 2000 years ago [4–8]. Since these livestock could not have been domesticated locally (their wild ancestors never lived in the southern hemisphere), researchers agree that they must have come from farther north, and different routes and chronologies have been proposed [2, 9–11]. But the question of how and in whose company livestock arrived has in the last two decades become a contentious topic. Did the animals indeed reach southernmost Africa with a sizeable migration of Khoe-speaking pastoralists [12–14]? Or were they traded down-the-line from one community to its neighbours, reaching the southern tip of Africa without accompanying herders[15–21]? Or indeed did one or more small-scale infiltrations of herders introduce livestock which subsequently diffused among innovative local foragers who thus became hunter-herders [22–24]? Related questions have been asked by linguists and geneticists about the role of Khoe-speakers in the original spread of livestock to southern Africaand whether the Khoe-speakers originally came from East Africa [25–30].

**Table 1:** The earliest directly dated livestock remains in southern Africa. Table arranged in chronological order from oldest to youngest dates.

Basin	Site	Lab No	Date BP	Sigma	Cal* 2 sigma	Comment	Reference
**Namibian Coastal**	Leopard Cave	Beta_270164	2270	40	390–170 BC	AMS date on Ovis aries (sheep) bone	[5]
**Namibian Coastal**	Leopard Cave	Beta-270163	2190	40	360–40 BC	AMS date on Ovis aries (sheep) bone	[5]
**Western Coastal**	Spoegrivier	OxA-3862	2105	65	350 BC-AD 90	AMS date on Ovis aries (sheep) bone	[7]
**Kalahari Drainage**	Toteng 1	Beta-1904888	2070	40	170 BC-AD 80	AMS date on Bos taurus (cow) bone	[6]
**Kalahari Drainage**	Toteng 1	Beta-186669	2020	40	60 BC-AD 140	AMS date on Ovis aries (sheep) bone	[6]
**Southern Coastal**	Blombos	OxA-4543	1960	50	20 BC-AD 240	AMS date on Ovis aries (sheep) bone	[4]

*Calibrated with Southern Hemisphere Atmospheric data in OxCal v3.10 [31, 32].

**Fig 1:**
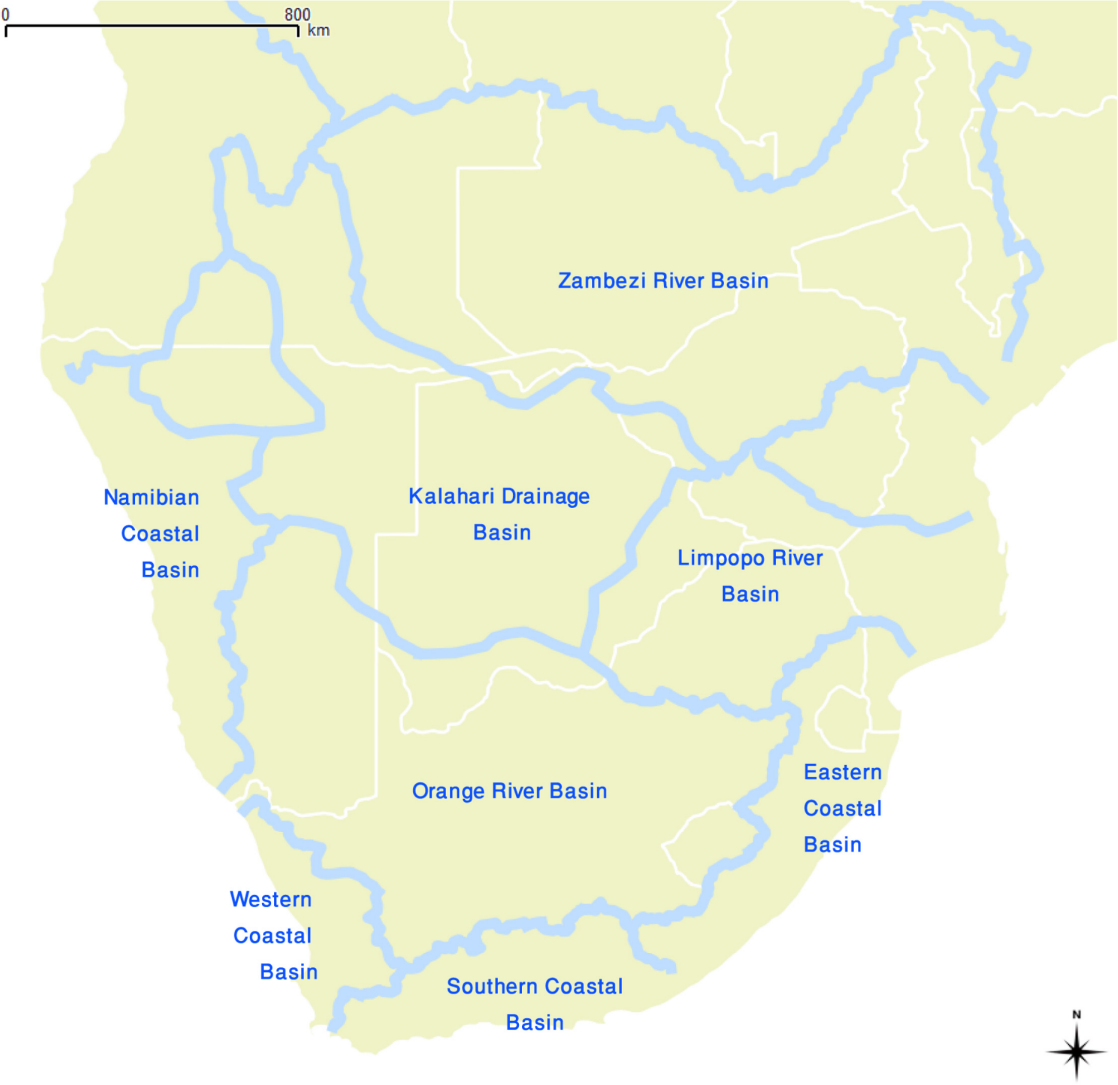
Map of sub-equatorial Africa. Country boundaries are shown withthin white lines and the major watersheds are shown with thick blue lines.

Importantly, it had been noted long ago that although innovations such as livestock and ceramic vessels appeared suddenly in the southern African landscape, stone tool (also known as lithic) sequences remained unchanged [15]. Indeed, for the most part they did remain unchanged; but at a few more recently excavated key sites there were significant changes in stone toolkits that shed new light on the question of how the first livestock arrived in southern Africa. In this paper, a new analysis of the distribution of key stone tool types, ceramic styles and early livestock remains in well-dated Later Stone Age sites of Africa south of the -10^th^ parallel shows that around 2000 years ago sheep and cattle were first infiltrated into southern Africa by small groups of hunter-herders on two separate fronts. In the extreme west, sheep were infiltrated southwards along the Atlantic seabord and as far as the southern tip of Africa by hunter-herders carrying a northern stone toolkit. Contemporary with this event, sheep and cattle as well as the art of pottery entered the middle Limpopo River Basin with a smaller infiltration of northern hunter-herders who crossed the watershed from the Zambezi River Basin (Fig 1). Rapidly, these innovations diffused among the Limpopo River Basin foragers, and then crossed the watershed westwards into the Kalahari Drainage Basin, this time with hunter-herders of the Limpopo River Basin lithic tradition who moved along the Makgadikgadi Pans and up the Boteti River Valley as far as Lake Ngami. A few centuries later Limpopo River Basin hunter-herders were responsible for the further spread of livestock southwards but we will leave the detailed discussion of that mid-first millennium AD event for a future paper.

The dispersion of new ideas, practices and products is a motor of societal change [33]. The important and measureable variables in such a dispersion are the time taken to adopt an innovation, the number of adopters at a given time, whether individuals or groups, and the channels through which innovations spread, as well as the types and numbers of boundaries which the innovation crossed [34]. Depending on a host of factors such as relative advantage, compatibility, complexity, trial-ability and observability, the innovation’s rate of adoption will vary [35], but not all of these factors are easily observed in our relatively coarse-grained archaeological data. When crossing boundaries, the diffusion of innovation becomes archaeologically more easily visible and a mosaic of mechanisms was identified by Zvelebil and Lillie [36] in the spread of farming into Europe. Some of these can also be observed in ancient southern Africa. They include well known mechanisms such as demic diffusion, which is the sequential colonization by random migration carried out by family groups over many generations, with daughter settlements budding off from the parental ones. An excellent example of demic diffusion is the Bantu-speakers’ migrations into and within southern Africa [37–44]. Leapfrog colonization by seafaring communities was important in the spread of farming across the Mediterranean Sea [45], but is perhaps less relevant in the early spread of farming and herding in southern Africa. Central to our study is the mechanism of infiltration, which refers to the gradual penetration of an area by small groups who entered subordinate positions in society, while ‘elite dominance’ is a similar mechanism but refers to an infiltrating minority that seized control. In Zvelebil and Lillie’s scheme, folk migration is the directional movement of a population from the old area of settlement to the new. Perhaps in part the spread of Bantu-speakers was by folk migration, but the first farmers in the Aegean islands provide a clearer archaeological example of this mechanism [46]. Finally, individual frontier mobility describes a mechanism in which individuals linked by friendship, partnership or kinship move between different communities across economic or cultural boundaries. William Barnett [47] proposed that this mechanism explains the spread of agriculture into the Mediterranean hinterland. Cultural practices such as exogamy and *Hxaro* gift exchange in the recent Kalahari [48] provided ample opportunities for such individual mobility across territories.

At the sub-continental scale of our study, demic diffusion and infiltration are important mechanisms for the diffusion of innovations. At the smaller sub-regional scales, however, individual mobility probably was a more important mechanism. Indeed all three of these mechanisms simultaneously can be active in the spread of innovations: Infiltration can form a bow wave ahead of demic diffusion, and individual frontier mobility can diffuse innovation ahead of the infiltrators. The boundaries between these three mechanisms can be vague and arbitrary. One can imagine them as ill-defined regions on a scalar continuum of diffusion of innovation with, at one extreme, innovations diffusing with the agency of many accompanying people (demic diffusion and folk migration), and at the other extreme no significant population displacement being involved in the diffusion of new traits (individual frontier mobility). In the grey middle ground of this continuum, infiltrationrefers to the diffusion of innovations by small groups of people. How many is many and how small is small? Different researchers would no doubt divide the continuum differently. In sociological and economic studies the spread of innovations can be objectively measured in time, scale and directionality [35]. In due time, archaeology will also be able to quantify, at least relatively, the speed, distance and size of population displacement involved in the spread of the first livestock into southern Africa.

### The Archaeological Data

We divide our region of interest into major drainage basins (Fig 1). Of interest here are the Zambezi River Basin (Z), the Limpopo River Basin (L), the Kalahari Drainage Basin (K), the Namibian Coastal Basin (N), the Orange River Basin (O) and the South African Western (W), Southern (S) and Eastern (E) Coastal Basins. Chronologically, we focus on events that took place around 2000 years ago. The first livestock and ceramic vessels, two innovations which were closely associated, appeared in southern Africa in the last few centuries BC (Table 1 and [49, 50]). Here, we divide our time span of interest in two phases. In the first phase we will look at the archaeological evidence from before the introduction of livestock into southern Africa, say the period from around 4000–2000 years ago; and in the second phase we consider the evidence from after their first introduction, a period from about 2000–1000 years ago although for now we are most interested in events that took place before the mid-first millennium AD.

In this space and time of interest, archaeological research coverage is of variable quality and quantity. We will concentrate on the Later Stone Age (LSA) archaeological sites and ignore the Iron Age sites which mostly post-date our main focus and mostly relate to Bantu-speaking farmers who apparently played no direct role in the earliest spread of livestock and ceramics into southern Africa [21, 49]. Different LSA archaeological entities have been named in the literature and we deal with the Nachikufan industries to the north of the Zambezi River and the Wilton to the south [15, 51–54]. The most recent proposal for southern African stone age terminology reserves Wilton for the period 8000–4000 years ago, and recommends the labels Final Later Stone Age and Ceramic Final Later Stone Age for more recent materials [55]. To simplify matters, we will restrict ourselves to the geographical and chronological terms, namely the major drainage basins and their principal Later Stone Age sites from our phases 1 and 2.

**Fig 2:**
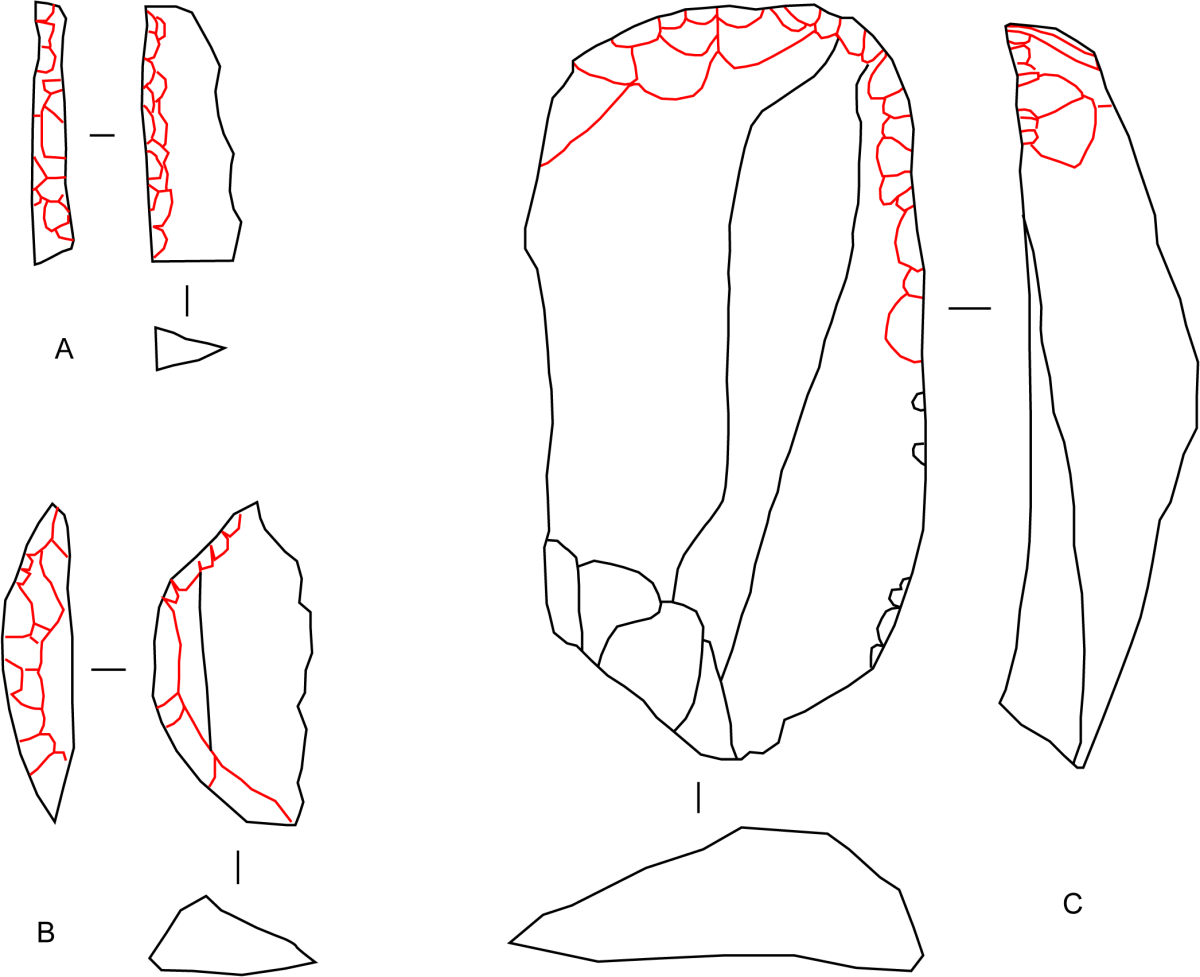
Illustrations of idealized lithic formal tools. (A, B) Backed tools. (C) Scraper. Each tool is shown in plan and side view, with a cross section beneath. Retouch scars are outlined in red. Backed tools of the southern African Later Stone Age usually are microlithic, which is to say less than 25 mm in maximum length. Scrapers can be larger but with repeated re-sharpening they become reduced to microlithic stubs before being discarded.

For stone tools we focus on two major classes. Formal stone tools refer to stone flakes which were retouched to re-sharpen an edge and/or to produce a desired and standardized form. The two major classes of formal tools under examination are scrapers (Fig 2C) and backed tools (Fig 2A and 2B). The former were mostly used for scraping animal hides, presumably in the production of leather [56–58]. There are several sub-types and significant size variations in Later Stone Age scrapers [15, 59], but at our scale of study we deal only with the general class of this tool and subsume all variants. Unlike scrapers, backed tools were retouched not primarily to shape and re-sharpen the business end of the tool, but to blunt the opposite (back) edge in order to facilitate hafting or to avoid cutting into the hand that used the tool. In Australia backed stone tools were used for a variety of purposes [60] and it is probable that the same is true for southern African backed tools [61]. As with the general class of scrapers, backed tools contain many sub-types but at our scale of study we are only concerned with the distribution of the general class and subsume almost all variants of backed tools. The exception is a particular tool called a backed scraper. Backed scrapers are relatively rare and have little effect on the results of our analysis regardless of whether we class them with scrapers or backed tools. However, because functionally they are thought to have served as scrapers, we prefer to count them with scrapers and not with backed tools.

It is important to note that scraping, cutting or piercing with stone does not require that the tool be retouched. As ethnographic and experimental studies show [62], freshly flaked stone without further ado will provide pieces with suitable edges for cutting, scraping or piercing, and Later Stone Age people mostly used un-retouched stone tools, sparing themselves the trouble of re-sharpening and/or trimming their stone flakes to a particular shape. At other times and in other places they invested a fair bit of effort into modifying their flaked stones to make them look just so, or they re-sharpened their tools to maximize their use-life. The point is that the modification of a stone flake by retouching is not purely for functional reasons: it is also a style of doing things and represents a culturally specific practice. We thus assume that the difference in the proportions of types of formal (that is to say retouched) tools is culturally patterned.

It has been known for some time that variations in the distribution of retouched scrapers and backed tools in southern African Later Stone Age sites produce significant chronological and geographic patterns, and several attempts had been made to explain their different distributions in functional terms [15, 52, 63]. Peaks in the proportions of backed tools have sometimes been interpreted as expressions of emerging social relationships, symbols that revealed cultural identities in times of increased formal exchange and gift-giving [64–67]. Others have emphasized that backed tools are portable, standardized and multifunctional tools and in one way or another enhanced efficient resource extraction, helping to offset the risk of uncertain environments [68–71]. Both views could apply in situations of high resource uncertainty, such as might obtain when populations move into new areas. Indeed, in Australia the proliferation of backed tools in certain times and places has been interpreted as a sign of migrations, reflecting risk reduction behaviour among newcomers in less predictable environments and/or where identity had to be clearly marked for new cultural contacts [72–74](. These ideas may also apply to southern Africa.

Our sample for analysis is restricted to Later Stone Age sites located in the drainage basins mentioned above. Site components that are associated with radiocarbon dates from the period between ca. 4000–1000 years ago, and that contain more than 20 formal stone tools are included in this study. A few sites and components that are not directly associated with dates, but whose age can be accurately estimated, have been included in our list. In the literature survey undertaken to date, a total of 123 archaeological site components fit our requirements. Many more Later Stone Age site components have been excavated in these drainage basins, but often they are not absolutely dated, have produced too few formal tools, or their stone tool counts have not been published. Such sites and components may provide supporting arguments but do not contribute to the quantitative analyses and the mapped data. Details of the sites used in our quantitative study are provided in Table 2.

**Table 2:** Our current data base of the sub-equatorial African LSA sites and components.

Basin_Site ID	SITE	layers	phase	SCRAPER%	BACKED%	FT	CAL DATES	References
E_7	BONAWE	1 a+b	3	100.0	0.0	24	400–40 BC	[75]
E_7	BONAWE	2	2	96.2	3.8	211	1090–800 BC	[75]
E_17	BORCHERS	3	2	50.0	28.6	196	1900 BC-AD 90	[76]
E_1	CLARKE'S	2	3	61.7	5.8	120	420–640 AD	[77]
E_1	CLARKE'S	3	2	54.5	3.6	55	750 BC-AD 20	[77]
E_2	COLLINGHAM	bsv3	3	31.0	12.1	58	70–340 AD	[78]
E_8	DIAMOND	1	2	66.4	11.8	110	1050–790 BC	[77]
E_12	DRIEL	older ash	3	58.3	0.9	108	220–430 AD	[79]
E_9	GEHLE	1	3	64.3	19.0	126	670–1390 AD	[80]
E_9	GEHLE	2	2	63.3	24.1	158	3090–2660 BC	[80]
E_6	INKOLIMAHASHI	4 & 5	3	35.6	6.7	45	400–1020 AD	[81]
E_6	INKOLIMAHASHI	7 & 8	2	52.0	32.0	35	1600–400 BC	[81]
E_5	KWATHWALEYAKHE	2	3	42.7	25.9	232	660–990 AD	[82]
E_5	KWATHWALEYAKHE	4	2	39.2	15.5	181	900–510 BC	[82]
E_14	MAQONQO	2	2	48.5	21.4	103	2020–1660 BC	[83]
E_3	MBABANE	4	3	54.6	19.0	163	430–890 AD	[84]
E_10	MGEDE	3	2	64.9	4.1	74	3350–2700 BC	[85]
E_4	MHLWAZINI	5_6	2	58.0	9.1	231	980–160 BC	[86]
E_15	MZINYASHANA	4	3	63.8	2.6	116	220–540 AD	[87]
E_15	MZINYASHANA	6	2	41.0	5.1	39	400–110 BC	[87]
E_11	NKUPE	3	2	13.1	2.0	343	1540–390 BC	[88]
E_13	SIKHANYISWENI	2	2	25.7	17.8	202	2460–2030 BC	[89]
E_16	UMBELI BELLI	2AL	3	55.1	14.3	49	810–1040 AD	[76]
K_5	MAHOPA	10_16	2	13.0	45.7	46	1690 BC-AD 240	[90]
K_7	TOTENG	40_140	3	40.0	32.5	40	170 BC-AD 670	[91]
K_7	TOTENG	140_200	2	15.2	81.8	33	900–410 BC	[91]
K_9	XAIXAI	9_18	3	7.2	59.8	97	400 BC-AD 530	[90]
K_9	XAIXAI	19_27	2	8.8	61.9	113	2300–1190 BC	[90]
L_2	BALERNO	dbg60-65_bra	3	71.5	10.0	1031	650–1160 AD	[92]
L_2	BALERNO	dbg >65	2	64.6	22.5	178	360–20 BC	[92]
L_1	BAMBATA	3a,b	3	33.9	40.2	174	360–60 AD	[93, 94]
L_1	BAMBATA	3c	2	37.6	44.4	117	2570–2200 BC	[93, 94]
L_4	JUBILEE	lyn-lyn4	2	51.6	22.8	184	1630–1310 BC	[95]
L_4	JUBILEE	b-cash	3	90.1	5.2	252	120–390 AD	[95]
L_7	MPHEKWANE	1_4	3	30.3	13.3	399	890–1160 AD	Sadr unpublished
L_7	MPHEKWANE	42102	2	23.2	10.8	203	800–200 BC	Sadr unpublished
L_9	OLIEBOOMSPOORT	1_5	3	86.1	7.2	746	250–880 AD	[96]
L_9	OLIEBOOMSPOORT	5_10	2	72.6	7.9	2388	420 BC-AD 70	[96]
L_11	RADIEPOLONG	1.16_27	2	61.8	14.7	34	1370–1000 BC	Sadr unpublished
L_12	THAMAGA	0–40	3	38.6	8.0	88	670–1050 AD	[97]
L_12	THAMAGA	40_60	2	38.0	33.8	71	3550–2750 BC	[97]
L_13	TSHISIKU	4_14	2	51.8	42.6	740	1500–910 BC	[98]
L_14	TULI LODGE	def	3	40.8	43.3	245	890–1030 AD	[99, 100]
N_19	AFFENFELSEN	complex a	2	29.4	55.6	870	1880–1530 BC	[101]
N_16	AUSTERLITZ	complex c	3	37.7	30.2	53	1–1000 AD est.	[101]
N_16	AUSTERLITZ	complex d	2	32.3	40.3	124	4500–1200 BC est.	[101]
N_23	BIG ELEPHANT SHELTER	pottery	3	40.8	21.7	120	550–1150 AD	[102]
N_23	BIG ELEPHANT SHELTER	pre-pottery	2	51.8	29.4	197	1440–410 BC	[102]
N_15	ETEMBA 14	complex c	3	30.6	48.4	62	360 BC-AD 60 est.	[101]
N_18	ETEMBA 2	complex b	3	35.1	19.3	57	10–260 AD	[101]
N_14	FACKELTRAEGER	complex b	3	43.5	34.8	46	360 BC-AD 60	[101]
N_14	FACKELTRAEGER	complex d1	2	37.7	32.1	53	1260–810 BC	[101]
N_2	FALLS ROCK SHELTER	5_9	3	49.3	50.7	67	200 BC-AD 340	[18]
N_2	FALLS ROCK SHELTER	1_4	2	33.8	66.2	68	3350–1450 BC	[18]
N_3	GEDULD	1_7	3	5.2	84.5	58	40 BC-AD 1380	[103]
N_22	HASENBILD	complex a	2	35.6	55.7	219	4500–1200 BC est.	[101]
N_17	MESSUM 1	complex b	3	17.4	39.1	23	630–870 AD	[101]
N_17	MESSUM 1	complex c	2	20.5	55.5	146	180 BC-AD 250	[101]
N_21	MESSUM 2	complex a	2	29.9	52.9	87	4500–1200 BC est.	[101]
N_13	N2000/2	all	3	19.4	77.4	31	120–350 AD	[104]
N_12	OMUNGUNDA 99/1	early cer	3	7.7	79.5	39	20–220 AD	[104]
N_12	OMUNGUNDA 99/1	micr lsa 1	2	13.8	51.7	29	410–170 BC	[104]
N_8	ORUWANJE 95/1	early cer	3	14.8	55.6	54	360 BC-AD 320	[104]
N_8	ORUWANJE 95/1	micr lsa 1	2	22.7	29.5	44	1220–410 BC	[104]
N_10	SNAKE ROCK SHELTER	3_5	2	52.6	47.4	38	3350–410 BC	[18]
N_24	STRIPED GIRAFFE	pre-pottery	2	55.7	27.1	70	4790–950 BC	[102]
O_26	BLOUBOS	all	3	70.5	29.5	332	130–400 AD	[105]
O_5	BLYDEFONTEIN	iii-iv	2	29.2	39.3	178	250 BC-AD 400	[106]
O_28	COLWINTON	3	3	86.1	1.6	445	60–330 AD	[75]
O_6	DIKBOSH	1,2	2	55.7	41.1	192	1440–1080 BC	[107]
O_6	DIKBOSH	1 to 3	3	69.3	15.8	101	250–650 AD	[107]
O_29	HOLKRANS	5_9	3	56.8	8.1	37	130–390 AD	Sadr unpublished
O_9	JAGTPAN7	3 and 4	2	26.1	47.8	23	790–410 BC	[108]
O_21	LIKOAENG	ii_ix	2	69.1	10.9	304	360 BC-AD 340	[109]
O_10	LIMEROCK	all	3	75.0	6.5	108	250–780 AD	[107]
O_19	MASITISE	3b	2	38.2	30.6	157	1870–1420 BC	[110]
O_27	RAVENSCRAIG	2	2	81.3	2.8	107	1390–1050 BC	[75]
O_12	RIVERSMEAD	i-iv	3	36.0	31.5	686	750 BC-AD 50	[111]
O_12	RIVERSMEAD	v-ix	2	45.3	20.8	298	910–370 BC	[111]
O_24	ROSE COTTAGE	a2	2	62.7	12.3	826	390–50 BC	[112]
O_23	SEHONGHONG	gap	3	80.6	3.2	62	260–980 AD	[113]
O_15	WITKRANS	0_30	3	82.4	15.0	187	540–670 AD	[107]
S_1	BOOMPLAAS	BLD	3	76.6	2.6	231	50 BC-AD 260	[114]
S_9	BYNESKRANSKOP	2	2	33.3	7.4	108	1780–1490 BC	[115]
S_10	DIE KELDERS	6_12	3	8.9	58.9	56	250 BC-AD 350	[116]
S_2	HAVENS	bed + pos	3	79.2	0.0	24	670–940 AD	[117]
S_2	HAVENS	oga	2	90.6	3.1	32	2000–1 BC est.	[117]
S_7	HIGHLANDS	iii	2	49.3	39.4	71	1980–1690 BC	[118]
S_3	KABELJOUS	kbl units	2	12.8	5.1	39	770–380 BC	[119]
S_6	MELKHOUTBOOM	caf	2	66.7	7.1	99	1260–800 BC	[118]
S_8	WILTON	2b	2	71.9	15.6	32	550 BC-AD 50	[120]
W_10	DEFLATION HOLLOWS	24 sites	2	47.8	19.2	8030	3000–1500 BC est.	[121]
W_21	FARAOSKOP	2	2	49.1	3.6	55	3330 BC-AD 240	[122]
W_16	KASTEELBERG A	all	3	10.0	0.0	20	70–1400 AD	[123]
W_5	KASTEELBERG B	12_16	3	19.0	0.0	21	660–1140 AD	[123]
W_6	KASTEELBERG C	bot	3	32.0	49.5	97	360 BC-AD 20	[123]
W_7	KASTEELBERG G	1_21	3	44.2	15.6	77	390 BC-AD 1180	Sadr unpublished
W_7	KASTEELBERG G	22_42	2	44.0	24.0	50	2940–1730 BC	Sadr unpublished
W_27	KN6-3C	upper	2	23.2	45.7	138	1890–1490 BC	[124]
W_25	MS3	all	2	9.1	81.8	22	1210–820 BC	[125]
W_8	PANCHOS	3_7	2	66.7	8.8	57	2030–510 BC	[126]
W_23	PN2009/001	all	2	72.0	15.9	107	520–340 BC	[125]
W_20	RENBAAN	AD	3	38.9	2.8	36	20–340 AD	[127]
W_24	SK2001/025	area f	3	48.1	25.9	27	80 BC-AD 100	[125]
W_24	SK2001/025	a-d	2	70.0	15.0	40	560–40 BC	[125]
W_11	SPOEGRIVIER CAVE	1_5	3	0.0	70.0	20	10–970 AD	[128]
W_11	SPOEGRIVIER CAVE	6_16	2	33.0	33.0	94	2030–1630 BC	[128]
W_12	STEENBOKFONTEIN	1_3	2	51.0	6.7	104	930–40 BC	[129]
W_13	TORTOISE CAVE	4_9	2	53.9	16.9	154	2700–1620 BC	[130]
W_19	VP SURVEY	18 sites	3	21.3	4.3	47	60–999 AD	[131]
W_19	VP SURVEY	13 sites	2	35.7	8.7	126	1729–44 BC	[131]
W_15	WITKLIP	3	3	22.6	14.3	84	80–820 AD	[132]
W_15	WITKLIP	4	2	48.6	18.9	37	1410–1050 BC	[132]
Z_1	CHAMINADE	ch3-ct	2	10.2	88.9	108	1870–1490 BC	[133]
Z_14	KANDANDA	4	2	60.0	12.0	50	2300–1250 BC	[134]
Z_3	LEOPARDS HILL	30–50	2	36.5	55.3	85	1960–800 BC	[51]
Z_5	MAKWE	6_4	3	1.7	98.1	1342	50–1230 AD	[135]
Z_5	MAKWE	3_1	2	4.9	95.0	903	3970–2550 BC	[135]
Z_6	MUFULWE	iii	3	12.5	67.3	104	260–540 AD	[136]
Z_9	NACHIKUFU CAVE	brown earth	2	17.4	70.4	1240	5700–2600 BC	[51]
Z_8	NAKAPAPULA	3	3	26.4	37.5	72	670–1140 AD	[137]
Z_8	NAKAPAPULA	5	2	20.8	43.8	48	1740–1290 BC	[137]
Z_7	THANDWE	1_6	3	2.1	97.9	282	770–1390 AD	[135]

Table arranged in alphabetic order of basin (initial letter) and then site name.

## Results

**Fig 3:**
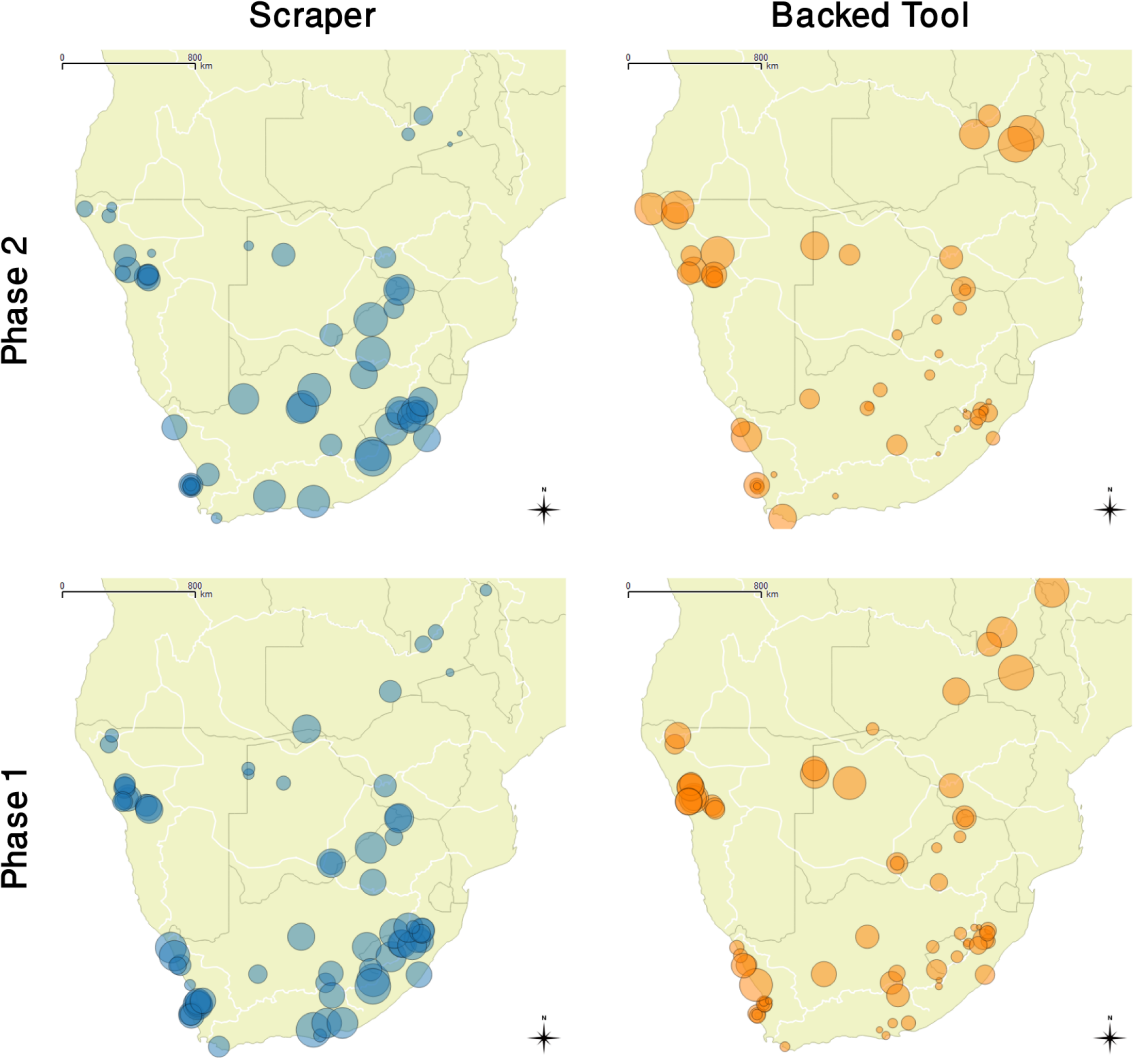
The distribution of scraper-rich and backed-rich assemblages in phase 1 and phase 2. The sizes of the circles reflect the percentage of scrapers/backed tools in the assemblage of formal tools at each site. In the background, thin dark lines show national boundaries and the thin white lines indicate the watersheds between basins.

In phase 1, backed-rich toolkits were dominant in the Zambezi River Basin, the Kalahari Drainage Basin and in the northern part of the Namibian Coastal Basin (Fig 3). Although relatively few sites from these areas passed the stringent requirements to be included in our analytic data base, the rejected site components (e.g., those not securely dated or with less than 20 formal tools, or incompletely published [104, 135, 138–142])echo and confirm the general impression that the phase 1 northern assemblages displayed a backed-rich lithic tradition. An anomalous cluster of (infiltrated?) scraper-rich toolkits are confined to the upper reaches of the Zambezi River Basin [134].

Phase 1 scraper-rich toolkits predominate in the Limpopo River Basin, the Orange River Basin as well as the Eastern and Southern Coastal Basins of South Africa. This pattern was also evident in the few thousand years preceding our phase 1 so a scraper-rich toolkit can be seen as a cultural emblem of southern Later Stone Age populations. There are a few interesting exceptions. The southern backed-rich toolkits found in phase 1 of Bambata Cave (Limpopo Basin), Jagt Pan and Blydefontein (Orange Basin) might represent small enclaves of the northern population from early (pre-livestock) infiltrations into the south. In fact, given that the dates from Bambata Cave are close to the phase 2 boundary (see Table 2), its backed-rich toolkit might actually relate to the introduction of the earliest livestock.

In the central Namibian Coastal Basin, scraper-rich as well as backed-rich assemblages are present, but the two are spatially discrete. As Wadley [102] noted, in Namibia the sites around the Brandberg contain backed-rich toolkits and resemble assemblages from farther north, while 100 km to the south and east, the sites around the Erongo Mountains with their scraper-rich toolkits resemble the southern Wilton assemblages. Unfortunately, none of the published LSA site reports for southern Namibia provide detailed lists of stone tools so they cannot yet be included in our data base. In the Western Coastal Basin of South Africa, only two sites, MS3 and KN6-3C, show a clear dominance of backed tools in phase 1 and they are both located in the northern parts of this basin, in today’s arid Namaqualand. Their associated dates (Table 2) suggest an infiltration much earlier than the introduction of livestock: in fact they may represent remnant backed-rich communities from the Mid-Holocene Altithermal (8000–4000 years ago) in Namaqualand [143]. It seems that warmer and drier periods in Namaqualand correlate with a southward shift of the boundary between the northern and southern lithic traditions. The first livestock here arrived with one of these northern advances.

At the interface of phases 1 and 2, in the last centuries BC, livestock simultaneously breached the north-south boundary in two different locations; along the Atlantic seaboard and from the Zambezi into the middle reaches of the Limpopo River Basin. In the Namibian Coastal Basin, the stone toolkits from the Brandberg northwards remained backed-rich into phase 2 (Fig 3). Most of the phase 2 stone toolkits around the Erongo Mountains remained as scraper-rich as they were in phase 1. But the boundary between the two traditions became less clear in phase 2 and the earliest ceramic vessels in this area sported decorations not unlike those found in the extreme north of this Basin [18, 101, 103, 104].The site of Geduld [103] which is on the same latitude as the Brandberg, was backed-rich in phase 1 and remained so in phase 2, but it now contained early evidence for sheep as well as the northern style of ceramics. The site of Leopard’s Cave in the Erongo Mountains contains the earliest dated sheep bones in southern Africa but its small collection of excavated lithics unfortunately yielded no formal stone tools and only a handful of undiagnostic potsherds [5].

Further south, in the relatively warm period at the dawn of phase 2, few dated sites are known from Namaqualand but at the site of Spoegrivier Cave the phase 1 scraper-rich assemblage was replaced with a backed-rich one, accompanied with sheep bones and ceramic vessels [128]. One of the Spoegrivier sheep bones produced the second oldest secure date for livetsock in southern Africa [8]. A few other phase 2 site components in the Western Coastal Basin such as Kasteelberg C [123], Bakoond [144], Reception Shelter [145] and Buzz Shelter [125] contain a backed-rich lithic assemblage, but except for Kasteelberg C they all yielded too small a sample of formal tools to be included on our maps. They nevertheless help confirm the idea of an infiltration by small groups bringing with them the northern lithic tradition. A good indication of the scale of this infiltration is provided by the fact that from over a hundred phase 2 sites recorded in the Western Coastal Basin during excavations and large scale archaeological surveys [121, 125, 146, 147], only a handful contain a formal tool assemblage wherein backed pieces are more numerous than scrapers. The large majority include the same scraper-rich toolkit as in phase 1.

Die Kelders Cave [116] at the west end of the Southern Coastal Basin is the farthest south that we can trace the infiltration of backed-rich toolkits. This site was not occupied in phase 1, but its early phase 2 occupation contains a backed-rich toolkit and sheep bones as well as an excellent collection of thin-walled black, highly burnished, mineral tempered pots. About 150 km further east in the Southern Coastal Basin, the site of Blombos produced another of the earliest sheep remains [4], but its small excavated LSA lithic collection contains only eight formal tools, none of which are scrapers or backed tools [148].

Ceramic vessels probably first reached the sites in the Western and Southern Coastal Basins along with the earliest sheep, from the Namibian Coastal Basin: The very early, perhaps locally invented fibre-tempered pottery typical of phase 2 in the Orange River Basin [49, 149] is all but absent on western and southern coastal sites. In the Western and Southern Coastal Basins, the innovation of pottery seems to have diffused more rapidly than the idea of herding because we find mineral-tempered potsherds on many of the scraper-rich phase 2 sites, but very few have yielded sheep bones that are clearly older than the mid-first millennium AD. The mid-first millennium AD increase in livestock in Western Coastal Basin sites such as at Kasteelberg A and B [123] relates to a separate infiltration (or demic diffusion?) event that originated in the Limpopo River Basin and which will be described in a future publication.

On the other front, the first sheep probably arrived in the Limpopo River Basin with a smaller scale infiltration of herders from adjacent areas in the Zambezi River Basin. We have already seen that a backed-rich toolkit appeared at Bambata Cave in the last few centuries BC. In the first few centuries AD, Bambata Cave and Tuli Lodge both show a backed-rich toolkit. The former includes sheep bones which unfortunately remain undated. Both sites contain a type of thin-walled mineral tempered pottery known as Bambata ware. Bambata ware diffused throughout the upper Limpopo River Basin among local scraper-rich communities of LSA hunter-gatherers and isolated sherds of Bambata pots are found as far as Manyana in south-eastern Botswana and Jubilee Cave near Pretoria [150, 151]. In the Limpopo River Basin, livestock seem to have been adopted by local communities because early in phase 2 some of them moved westwards into the Kalahari Drainage Basin, taking their scraper-rich toolkit plus Bambata pottery and livestock via the Makgadikgadi Pans and the Boteti River valley as far as Lake Ngami at the southern tip of the Okavango Delta. Bambata pottery is found at several LSA sites along this route [152, 153] but unfortunately none have had their lithic finds published in detail. Only from the well-dated site of Toteng 1 near Lake Ngami [91] do we have quantified information about the stone tools. In phase 1, Toteng contained a northern backed-rich toolkit typical of the Kalahari Drainage Basin LSA sites. With the arrival of livestock at this site, Bambata pottery and a scraper-rich toolkit also appeared. The early phase 2 sites further north and west from Toteng, such as those in the Tsodilo Hills and near the Dobe waterhole [90, 138–142], contain neither livestock nor Bambata pottery, and their toolkits remained backed-rich into phase 2. These bits of evidence indicate that the livestock and Bambata ware at Toteng did not come from the north and west but from the east, brought by hunter-herders from the Limpopo River Basin.

**Fig 4:**
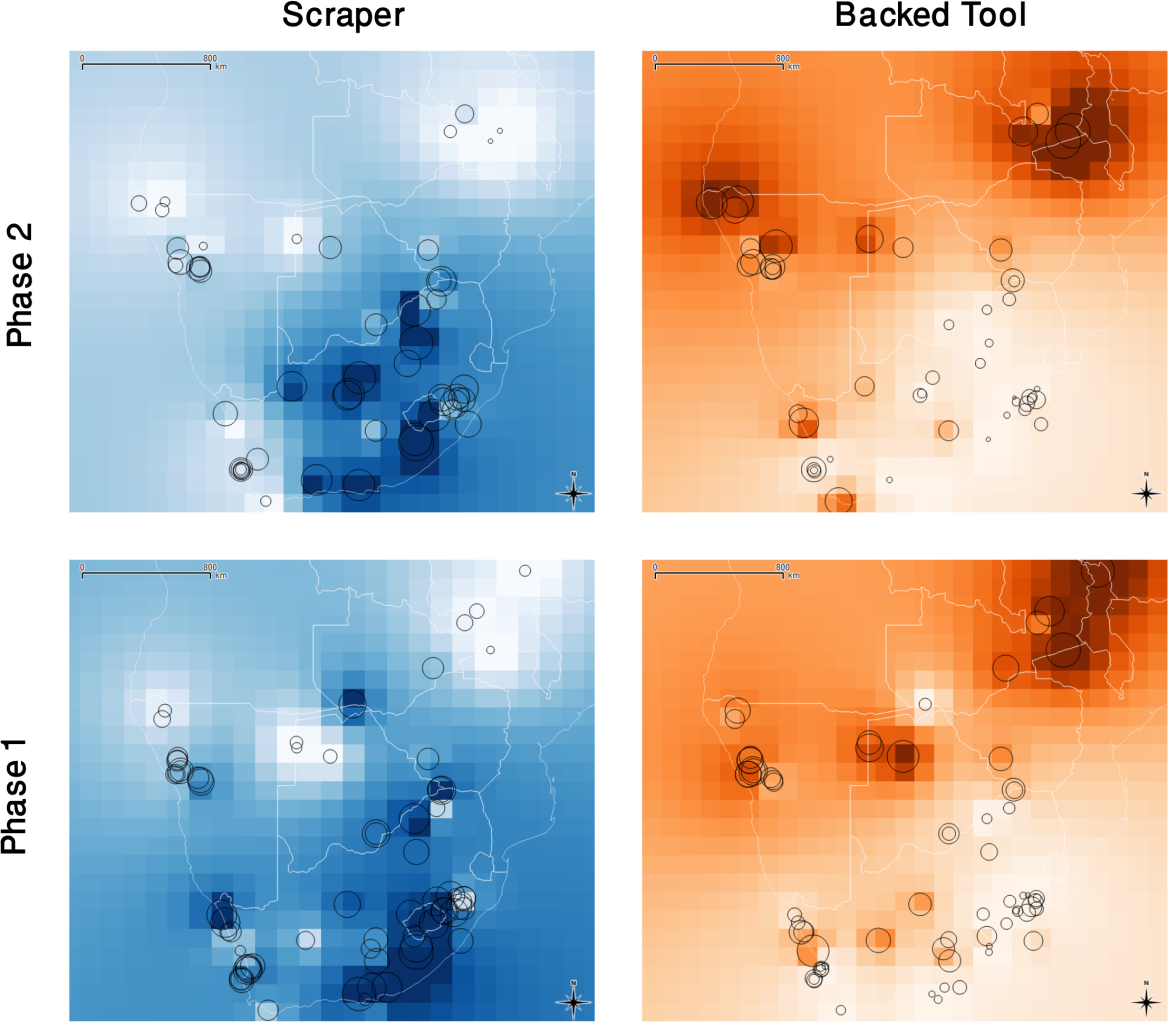
Inverse Distance Weighted interpolations of scraper and backed tool distributions in phases 1 and 2. Darker cells indicate higher percentages of the tools in question. In the backgrounds of each image, the site circles from Fig 3 as well as the national boundaries are faintly visible.

Considering the sub-continental scale, our data points for this study are few but we can use them as a basis for interpolating the distributions of scraper-rich and backed-rich toolkits in sub-equatorial Africa over the period of interest. Using QGIS software (version 2.8.2) and its standard plug-ins [154], a routine inverse distance weighting interpolation of the data was carried out. The study area was gridded in 27 columns and 25 rows and the interpolation was run with a distance coefficient of p = 2. This produced the maps shown in Fig 4. The interpolated maps in the right hand column show the northern concentration of backed-rich toolkits: in phase 2 (top row) isolated enclaves of backed-rich toolkits reached the south coast. Importantly however, backed-rich enclaves were also present in the south in the first phase suggesting that small scale movements of populations in this landscape were not a unique anomaly necessarily tied to the spread of livestock. In the left hand column of Fig 4, the interpolated distribution of scraper-rich toolkits shows the predominantly southern concentration of this trait. In phase 2 the scraper-rich toolkits became less prominent in the north and west, while becoming more conspicuous in the Limpopo and Orange River Basins in the north-central portion of South Africa. This may be related to the mid-first millennium AD event that brought stylistic and functional elements of Bambata ware to the south and west coasts. The similarity in the extent of this interpolated phase 2 scraper-rich patch in north-central South Africa and the distribution of so-called Khoekhoe (also known as geometric) rock art is [155] worth noting.

## Discussion and Conclusion

In this paper we have seen that the sub-equatorial African Later Stone Age sites of the last two millennia BC, our phase 1, can be divided into a backed-rich tradition in the north and a scraper-rich one in the south. Others had already noted this division [15, 54, 99, 102]. What is new here is that the appearance of the earliest livestock and pottery in southern Africa can be linked to the northern backed-rich tradition on two separate infiltration fronts. On the Atlantic seaboard during the last few centuries BC, one or more small groups of northern backed-rich stone toolkit makers infiltrated livestock and thin-walled mineral tempered ceramic vessels as far as the southern tip of Africa. Farther east, a smaller infiltration of northerners initially introduced livestock and probably Bambata pottery across the Zambezi/Limpopo watershed. From there, Bambata Ware diffused up the Limpopo River basin and some local hunter-gatherers with their scraper-rich toolkit adopted livestock and Bambata ware, taking them westwards across the Limpopo/Makgadikgadi watershed and establishing enclaves as far as Lake Ngami, where they replaced the local backed-rich lithic tradition. But to the north and west of Lake Ngami, hunter-gatherers continued to make backed-rich toolkits and adopted neither the Bambata Ware nor livestock herding at this time. Later, in the mid-first millennium AD, Bambata Ware disappeared with the encroachment of the Iron Age way of life, but some of its stylistic and functional traits reappeared on the western and southern coast of South Africa.

These conclusions, based on faunal remains, stone tool types and pottery styles, echo several of the latest findings from genetic studies. Two studies of autosomal DNA diversity in southern African Khoisan populations divide those in the northwest Kalahari Basin (Ju speakers:! Xun and Ju|'hoansi) from those in the southeast (Tuu and Khoe speakers: Karretjie, ≠Khomani, and Nama), and date this split to the last 30,000 years [156, 157]. This genetic divide matches the split between our northern and southern lithic traditions quite well. However, in other analyses the division of the NW and SE Kalahari groups, based on mtDNA, is not so clear-cut [158]. This difference in clarity of the divide seen in autosomal versus mitochondrial DNA, between the male and the female lineages, perhaps indicates a higher rate of individual frontier mobility among females due to exogamy and patrilocality among the Khoisan.

There is also some genetic evidence to support the linguistic hypothesis [27] that the first herders of southern Africa were Khoe-Kwadi-speakers who originated in East Africa. The distribution of Y chromosome haplogroup E-M293 suggests a movement of people from Tanzania to southern Africa before the Bantu migration [30]. Autosomal data as well as a lactase persistence allele [156, 157] provide evidence of some shared ancestry between the Khoe-speakers, such as the Nama and Shua, with East African populations. But the Nama show much genetic similarity with the southern San groups such as the ≠Khomani and Karretjie and only share a small genetic ancestry with East African groups [157]. This East African component is also present at lower levels in the ≠Khomani and Karretjie, but is extremely rare in the! Xun, the Ju/'hoansi, and the /Gui and //Gana [157]. The scale of admixture suggests that the East African connection was not due to mass population movement, but rather indicates movement of small groups perhaps commensurate with what we have called infiltration. According to Barbieri et al. [158], the presence of mtDNA haplogroups L0d and L0k lineages in the Khoe-speaking populations indicates contact with local San foragers. Admixture of San with the immigrants did not leave evident traces in the maternal genetic material of the local San, which can suggest that the infiltration from East Africa was mainly by male herders. The autosomal and the mtDNA data reveal a highly complex pattern of prehistoric population movements. Like the archaeological evidence, they seem to argue against a single, large-scale migration of a pastoralist population prior to the arrival of the Bantu-speakers.

The diversity of the infiltration events can be gauged to some extent by examining the types of genetic admixture in Khoisan populations. A potential East African genetic candidate is mtDNA haplogroup L5, common in East Africa and present exclusively in the Shua and Tshwa at 5% and 18%, respectively [158]. These two populations currently inhabit the eastern side of the Kalahari Drainage Basin, near the Makgadikgadi Pans and the Boteti River. L5 is notably absent in the Okavango and Nama populations who are today found, respectively, around the Okavango Delta and in central and southern Namibia. The Nama show the clearest signal of ancestry with East Africa in their autosomal (male lineage) data [156, 157]. This mixture of genetic signals could indicate that the Shua and Tshwa may have acquired L5 from females crossing the frontier individually into the eastern parts of the Kalahari Basin, while the Nama on the western edge of the Kalahari Basin may have been infiltrated directly by the East African males. Although the chronology of these events is not precisely indicated in the genetic data, they do not in general contradict the idea that livestock and ceramic vessels may have reached the Limpopo River Basin mainly through a process of individual frontier mobility and that the foragers in the western coastal areas received sheep and pottery mainly in a process of infiltration by northerners. But, it is also possible that high levels of contact with local foragers in the maternal line erased any original signal of East African maternal ancestry in the Nama [158].

The scale of the infiltrations can perhaps be gauged by examining the proportions of genetic admixture in Khoisan populations. To provide a basis for comparison, it is worth noting that the Bantu migrations, which are clearly evident in the archaeological record are also strongly recorded in the genetic data. In south-western Angola, among some of the most admixed Bantu-speaking populations of southern Africa, the patterns of lineage sharing and admixture estimates suggest that around 75% of mtDNA variation can be traced back to West-Central Africa, which indicates a significant population migration and not a minor infiltration [39]. In general, the maternal genepool of the Bantu-speaking populations of southern Africa is very homogenous [37], again indicating that a coherent and large population was involved in the migration that brought them into southern Africa.

In contrast, the diversity of the mtDNA and autosomal genepool among the Khoe-speakers indicates a much more complex series of small-scale population movements, perhaps of the scale that we have classified in the archaeological record as infiltrations and individual frontier mobility. Of the different East African genetic components, for example, the Afro-Asiatic component is largest in the Nama where it only reaches 11%; and the East African ancestry does not exceed 6% in the other southern African Khoisan groups[29]. The Nama were found to have high levels of the -14010*C Lactose Persistence allele. This allele occurs in 15 of the Khoisan populations and four of the Bantu-speaking groups, at an overall frequency of 7.4% [159]. It was first reported in Kenya and Tanzania at overall frequencies of 28% and 32%, respectively, but is rare or absent in other populations. The -14010*C allele occurs at significantly higher frequency in the Khoe-speakers (11.3%) than in Tuu-speakers (2.4%), Kx’a-speakers (4.1%), or Bantu-speakers (3.9%). These results suggest that the -14010*C allele was brought to southern Africa from East Africa by herders who either interacted predominantly with Khoe speakers or perhaps even spoke languages which were ancestors of the Khoe languages [159]. But the proportion of East African input into the genetic composition of southern African Khoisan remains relatively low and is not evenly distributed among all Khoe-speakers: The genetic data suggest that the Nama originate from a southern San group with some introgression from an East African group [157]. There is also west Eurasian ancestry among the Khoisan some of which came via East Africa; the highest levels of this are found in the Nama, where it reaches 14% but that also includes the impact of recent colonialism [160]. As an extreme example, the input of E-M293 haplotypes from East Africa could have been achieved by as few as four male individuals [30]. All this favours the model of multiple, separate small infiltration events rather a coherent large-scale population migration as the motor for the introduction of East African traits into southern Africa.

The chronology of all these genetic contributions is relatively imprecise. Estimates based on shared E-M293 haplotypes indicate that gene flow between eastern and southern African populations most likely occurred between 1200 and 2700 years ago (standard error bounded by 40–5000 years ago [30]). The admixture event which introduced Eurasian genetic traits, and which had the largest demographic impact in Khoisan populations that speak Khoe–Kwadi languages, can be dated to ∼900–1800 years ago [160]. And the analyses of the LCT region and genome-wide data among southern Africans show that the pastoralist Khoe originate from a San group that adopted pastoralism, with introgression from an East African Afro-Asiatic group that migrated south prior to 1300 years ago [29]. Using the Maasai and Ju|’hoansi as potential parental populations to the Nama, an admixture date of 1143 ± 74 years is indicated. Using the Afar, Amhara, and Tigray instead of Maasai, the admixture dates would be somewhat older around 1255 years ago [29]. It is interesting to note the diversity of these chronological estimates, and that many are too recent to correspond to the earliest infiltrations of livestock into southern Africa. All this suggests that many separate infiltration events brought East African cultural, economic and genetic traits into southern Africa over a long time span. With the help of large scale patterns in the distribution of stone toolkits, ceramics and faunal remains, we have been able to isolate two of the events which infiltrated the first livestock into southern Africa.
